# Antibacterial and Cytotoxicity Evaluation of New Hydroxyapatite-Based Granules Containing Silver or Gallium Ions with Potential Use as Bone Substitutes

**DOI:** 10.3390/ijms23137102

**Published:** 2022-06-26

**Authors:** Kamil Pajor, Anna Michalicha, Anna Belcarz, Lukasz Pajchel, Anna Zgadzaj, Filip Wojas, Joanna Kolmas

**Affiliations:** 1 Department of Analytical Chemistry, Chair of Analytical Chemistry and Biomaterials, Medical University of Warsaw, Faculty of Pharmacy, 02-097 Warsaw, Poland; kamil.pajor@wum.edu.pl (K.P.); lukasz.pajchel@wum.edu.pl (L.P.); 2 Chair and Department of Biochemistry and Biotechnology, Medical University of Lublin, 20-093 Lublin, Poland; anna.michalicha@gmail.com (A.M.); anna.belcarz@umlub.pl (A.B.); 3 Department of Environmental Health Sciences, Medical University of Warsaw, Faculty of Pharmacy, 02-097 Warsaw, Poland; anna.zgadzaj@wum.edu.pl (A.Z.); filip.wojas@gmail.com (F.W.)

**Keywords:** silver, gallium, calcium phosphates, biomaterials, antibacterial activity

## Abstract

The aim of the current work was to study the physicochemical properties and biological activity of different types of porous granules containing silver or gallium ions. Firstly, hydroxyapatites powders doped with Ga^3+^ or Ag^+^ were synthesized by the standard wet method. Then, the obtained powders were used to fabricate ceramic microgranules (AgM and GaM) and alginate/hydroxyapatite composite granules (AgT and GaT). The ceramic microgranules were also used to prepare a third type of granules (AgMT and GaMT) containing silver or gallium, respectively. All the granules turned out to be porous, except that the AgT and GaT granules were characterized by higher porosity and a better developed specific surface, whereas the microgranules had very fine, numerous micropores. The granules revealed a slow release of the substituted ions. All the granules except AgT were classified as non-cytotoxic according to the neutral red uptake (NRU) test and the MTT assay. The obtained powders and granules were subjected to various antibacterial test towards the following four different bacterial strains: *Staphylococcus aureus*, *Staphylococcus epidermidis*, *Pseudomonas aeruginosa* and *Escherichia coli*. The Ag-containing materials revealed high antibacterial activity.

## 1. Introduction

A significant increase in the development of biomaterials for use in bone disease treatment has been recorded in recent years. One of the main reasons is the increasing number of orthopaedical surgeries and the need to replace bone tissue with an appropriate multifunctional biomaterial [1,2,3,4,5].

Currently, materials are being sought that can act as carriers for delivering drugs to the bone as a poorly vascularized tissue. Particular importance is attached to the administration of antibacterial agents (antibiotics) in this way, due to the high risk of potential bacterial infection during surgery, known as surgical site infections (SSIs) [6,7,8,9,10].

Calcium phosphates (CaPs) are used to a great extent in orthopaedic surgery and dentistry, in the form of cements, scaffolds, granules or coatings [11,12,13]. Among the CaPs, synthetic hydroxyapatite (HA), with the formula Ca_10_(PO_4_)_6_(OH)_2_, can be distinguished, due to its beneficial properties, such as its similarity to bone mineral, bioactivity, osteoconductivity and non-toxicity [14,15,16,17].

It is worth mentioning that HA can be easily modified by various ionic substitution, in order to obtain additional biological or physicochemical properties [13,15,17]. For example, the incorporation of carbonates (CO_3_^2^^−^) into the HA structure leads to an increased solubility of material and a great tendency to nanocrystallinity, whereas substitution with fluorides (F^−^) causes better thermal stability of HA [18,19,20,21,22]. Moreover, a feature of HA that deserves attention is the ability to adsorb many biologically active substances; therefore, HA can potentially be used as a carrier for drugs [15,16,23].

Silver has been well known for its antibacterial properties for many years (its beneficial role in the treatment of infection dates back to at least 4000 B.C.) and its mechanism of action is one of the best understood [24,25]. Silver ions mainly affect thiol groups (-SH), present in bacterial proteins’ structure, by substituting hydrogen atoms and the arising of S-Ag binding. Such modifications in the protein structure of bacteria cells cause a denaturation, deactivation and malfunction of membrane pumps. As a consequence, membrane cells shrink and detach from the cell wall, then the content of the cell leaks outside the membrane and finally, the cell wall is torn apart [26].

As an antibacterial agent, silver is currently used in hospitals to reduce nosocomial infections in the treatment of burns and open wounds, but also in water cleaning systems [24,27]. Recently, silver has been identified as a promising agent for potential use in treating multidrug-resistant bacteria infections [28,29]. Many studies focusing on the synthesis of silver-substituted HA have been reported, which highlight its abilities in inhibiting bacterial growth and simultaneously intensifying osseointegration, consequently resulting in silver-doped HA being regarded as a very promising biomaterial [30,31,32,33].

Gallium ions also exhibit antibacterial activity; however, their use is not as common as silver. Their main advantage over silver ions is significantly lower cytotoxicity in higher concentrations with regard to human cells. The Ga^3+^ antibacterial mechanism of action is mainly based on the substitution of iron ions with gallium ions in the bacteria protein metabolism (“the Trojan horse strategy”), which causes the impairment of bacteria cell functions [26,34]. Moreover, there are many studies that outline the beneficial effect of gallium on bone tissue. It should be noted that Ga ions exhibit antiresorptive and antiosteoporotic properties, as well as antitumor, anti-inflammatory and immune suppressive properties [26,35,36]. As a result, HA doped with gallium can be used as biomaterial, which on the one hand enhances bone growth and on the other hand protects from bacterial infection [37,38,39,40]. In the available literature, the use of the aforementioned biomaterial as a drug delivery system is reported [41,42,43].

In the present work, the new bone substitutes, based on HA modified with Ag^+^ or Ga^3+^ ions as antibacterial agents, were prepared in the form of three types of granules. The obtained materials were subjected to physicochemical analysis, followed by cytotoxicity and antimicrobial evaluation.

## 2. Results

### 2.1. Chemical Structure and Elemental Analysis of the Synthesized Powders

**Figure 1:**
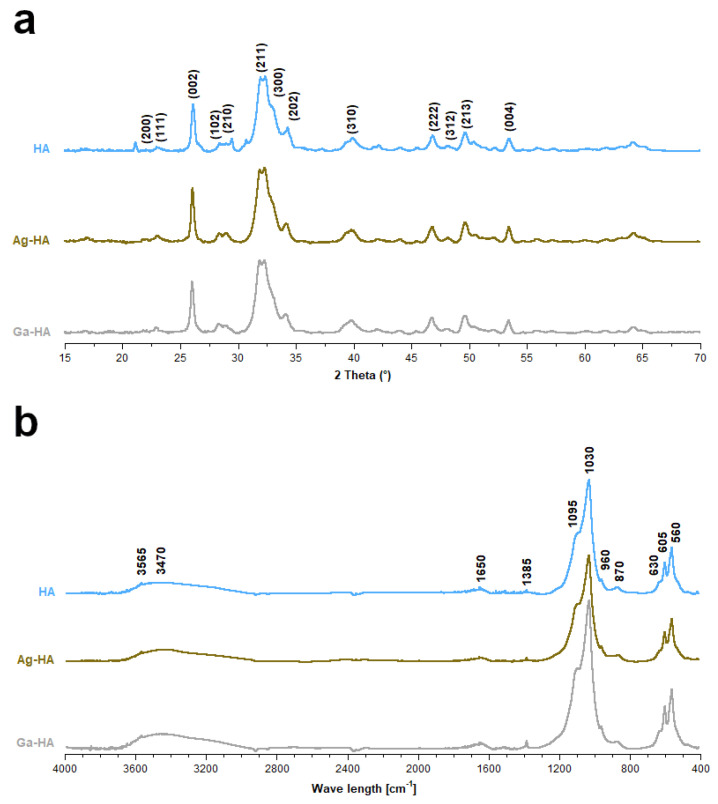
Powder X-ray diffractograms (**a**) and FT-IR spectra (**b**) of the synthesized powder samples.

The powder X-ray diffractometry (PXRD) patterns of the samples are presented in Figure 1a. The revealed reflections in all the diffractograms indicated that the materials are composed of hydroxyapatite. The obtained powders are homogenous, with no additional crystalline phase.

It is worth mentioning that the reflections were broad and poorly resolved, illustrating the poorly crystalline feature of the synthesized powders. In order to determine crystallite sizes along *c* and *a* axes, the Scherrer formula was used for the reflections at approx. 25.9° and 39.8°, respectively [44], which is as follows

(1) $$d=\frac{0.94\lambda }{\beta \cos\theta },$$

where

*d*—crystallite size (nm)
*λ*—wavelength of used radiation (nm)
*β*—full width at half maximum (FWHM) of the peak (radians)
*θ*—the diffraction angle of the corresponding reflection (°).

It was also possible to evaluate the crystallinity of the materials, using the following formula [45]:

(2) $$\chi ={\left(\frac{K}{\beta_{\left(002\right)}}\right)}^{3}$$

where

*χ*—the degree of crystallinity
*K*—constant (for hydroxyapatite it is equal to 0.24)
*β*_(002)_—full width in a half minimum (FWHM)for (002) reflection (°).

The calculated parameters are shown in Table 1. The values of the crystal dimensions confirm that apatitic crystals in all the samples were elongated along the *c* axis. The samples are nanocrystalline with a low degree of crystallinity.

The Fourier transform infrared spectroscopy (FT-IR) spectra presented in Figure 1b show a band corresponding to the apatitic chemical structure. One can observe three intensive bands within the range of 1095–960 cm^−1^ and two bands within the range of 605–560 cm^−1^, originating from the stretching and bending vibrations of phosphate P–O bonds, respectively. At approximately 3565 cm^−1^ and 630 cm^−1^, respectively, the stretching and libration vibrations of the characteristic structural hydroxyl groups can be observed. In addition, a wide band at approx. 3470 cm^−3^ (stretching) and a band at 1650 cm^−3^ (bending) confirm the presence of adsorbed water in the samples, which is common for the wet method. The slightly detectable band at 1385 cm^−3^ originates from the nitrate residues, while the band at 872 cm^−1^ comes from the carbonates [46,47,48,49].

The Ag^+^ and Ga^3+^ ion content in the Ag-HA and Ga-HA samples, respectively, were measured by the atomic absorption spectrometry (AAS) method. The elemental analysis shows that the dopant’s concentration is slightly lower than the theoretical value (0.54 and 0.45% for Ag and Ga, respectively), indicating only a partial substitution of these elements into the apatitic structure (see Table 1).

### 2.2. Ultrastructure, Porosity and Mechanical Strength of the Granules

**Figure 2:**
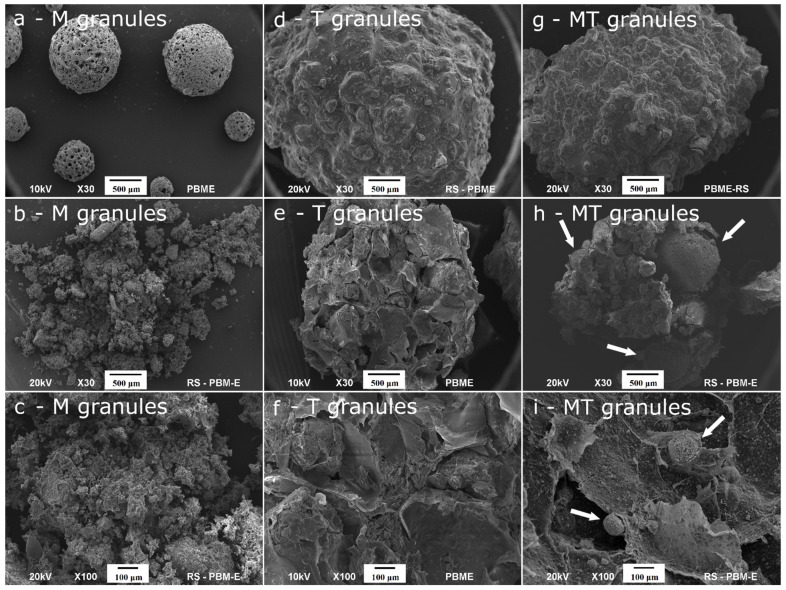
Representative SEM images of the samples: ceramic microgranules (AgM and GaM) (**a**–**c**); composite granules (AgT and GaT) (**d**–**f**); composite granules AgMT and GaMT (**g**–**i**).

The representative scanning electron microscopy (SEM) images of various types of granules are shown in Figure 2a–i. The ceramic AgM and GaM granules comprised micro-sized, regular spheres with an approximate diameter of 0.2–1 mm (see Figure 2a). The composite granules (AgT, GaT, AgMT and GaMT) were significantly larger and their average diameter was around 3.5 mm (see Figure 2d,g). As observed, all the samples revealed a porous feature; however, the outer surface of the ceramic microgranules seemed to be more porous than the outer surface of the composite, which in turn was rough and undulating. In Figure 2b,c,e,f,h,i, the cross-sections through all the types of granules are presented. It should be noted that the large number of macropores and mesopores in the internal structure of the granules can be observed, especially in the composite granules, which may indicate a high porosity of the materials. In addition, the cross-sections through the AgMT and GaMT granules show the microgranules, which were used during their preparation (indicated with white arrows in Figure 2h,i).

The porosity measurements using the mercury intrusion were only available for the composite granules (see Table 2). As observed, the total volume of pores and the degree of porosity is significantly higher for the AgT and GaT granules than for the AgMT and GaMT granules. It is worth mentioning that the specific surface area (SSA) of the pores is also better developed for the AgT and GaT granules, which is in accordance with the SEM results (see Figure 2d–f). The high SSA of these granules may be caused by their well-developed mesoporous structure. This is also an important factor in the case of the average diameter of pores; highly developed mesopores, as well as the presence of numerous macropores, may be the reason for a larger diameter of pores in the case of the AgT and GaT granules (see Table 2).

It should be noted that the average mechanical strength of the synthesized granules was vastly different in two analyzed types of granules (see Table 2). The AgT and GaT granules exhibited mechanical strength of around 35 N, which was approximately three times lower than the mechanical strength of the GaMT granules. This may be explained by the higher apparent density of the GaMT granules. Surprisingly, the AgMT granules showed significantly lower mechanical strength, together with great apparent density.

In the case of the ceramic microgranules (AgM and GaM), it was not possible to conduct an evaluation of the mechanical compressive strength and mercury intrusion porosimetry method, due to the very small size of the microgranules and their pores. Therefore, we decided to estimate the sizes of the pores using the SEM pictures (data not shown) and Olympus software measure IT.

According to this evaluation, ceramic microgranules AgM and AgT have two types of pores, including larger, irregular ones with a diameter of about 25 ± 3 µm and 15 ± 3 µm for AgM and GaM, respectively, and smaller, oval ones with a diameter of about 5 ± 2 µm.

### 2.3. Study of Silver and Gallium Ions Release from Granules

**Figure 3:**
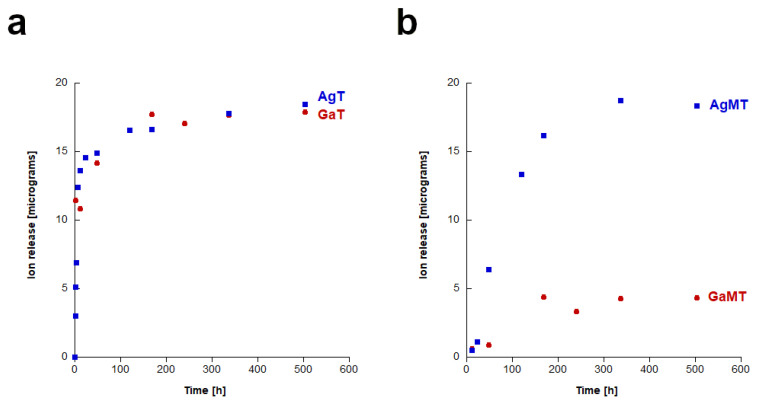
The results of the release study of silver and gallium ions from: AgT and GaT granules (**a**); AgMT and GaMT granules (**b**).

In Figure 3a,b, the results of the release of silver and gallium ions are presented. It can be observed that in all of the cases (for the “T” granules, as well as the “TM” granules), the amount of released silver ions was fairly low. The lowest release was observed for GaMT granules. It is also worth noting that in the case of the AgMT and GaMT granules, silver or gallium ions started to be released after a longer time than from the AgT and GaT granules, respectively. Regarding the AgT and GaT granules, the presence of the doped ions could be observed in the sample after only 1 h, whereas in the case of the AgMT and GaT granules, the ions were detected in the samples after 12 h. However, in the case of the silver-containing samples, this did not influence the final concentration of silver ions, which was similar in both cases.

### 2.4. Cytotoxicity Studies of Powders and Granules

**Figure 4:**
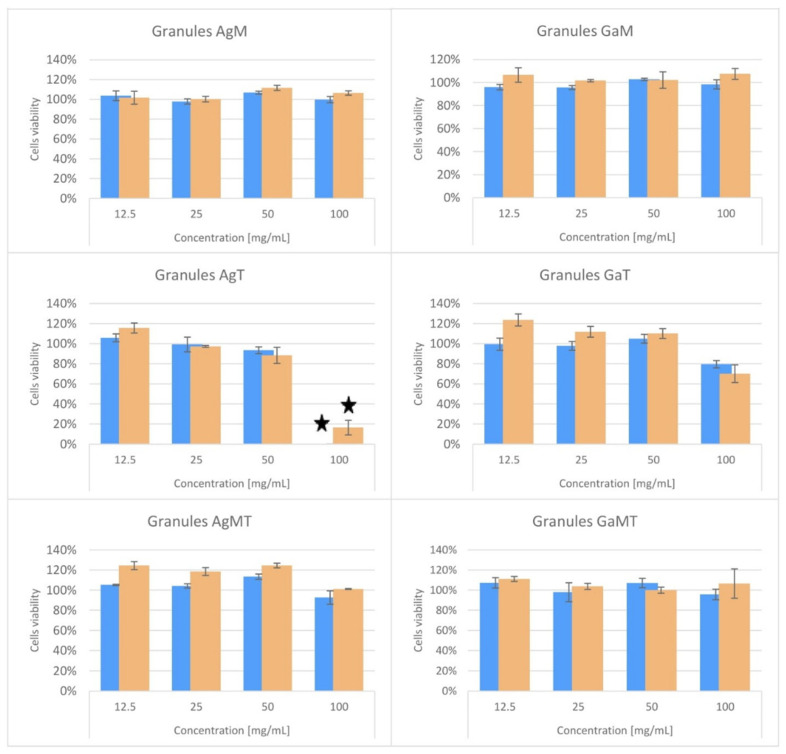
The NRU (blue bars) and MTT (orange bars) tests results obtained for granules in the whole range of tested extracts concentrations. Black stars indicate the decrease in the cells’ viability under 70%, which classifies the sample as cytotoxic.

Figure 4 and Figure S1 (in Supplementary Materials) show the results obtained in the cytotoxicity tests. As can clearly be observed, the BALB/c 3T3 cells viability or mitochondrial metabolic activity did not fall below 70% in comparison to the untreated control, using the AgM, GaM, GaT, AgMT and GaMT granules in the NRU; neither was this the case in the MTT test across the whole range of tested dilutions (Figure 4). Therefore, all these materials were classified as non-cytotoxic in both assays (Table 3 and Table 4). On the other hand, Ag-HA powder (see Figure S1) and AgT reduced 3T3 cells viability and enzymatic activity to below 70% after exposition to the undiluted extracts (100 mg/mL) and were classified as cytotoxic in both assays (Table 3 and Table 4). The Ga-HA powder was not classified as cytotoxic, according to the methodology of the NRU assay, but it significantly decreased the mitochondrial metabolic activity of cells treated with the undiluted extract (see Figure S1 and Table 3). However, in the first of the dilutions in the twofold dilution series, none of these samples (Ag-HA, Ga-HA powders and AgT) negatively affected the cell culture condition.

### 2.5. Results of Antibacterial Activity Studies

#### 2.5.1. Preliminary Studies

The pilot test of antibacterial activity was based on the migration of Ag and Ga ions from the prepared samples to the surrounding agar medium and inhibition of bacterial growth within the migration zone. The results obtained in this experiment showed that only the Ag-HA powder and porous AgT granules exhibited antibacterial activity (Table 5), confirming the release of Ag^+^ from the samples. Larger growth inhibition zones were observed for Ag-HA powder (11–13 mm) than for AgT granules (8–10 mm). This difference can be explained by the fact that the granules are composed not only of Ag-HA powder but also of sodium alginate and chondroitin sulphate; therefore, the content of Ag-enriched powder was lower in the granules. The lowest susceptibility to silver ions was noted with regard to the *E. coli* strain (Table 5). In the case of the AgM ceramic microgranules, no antibacterial activity was observed for all tested strains. This was expected, due to the low porosity of the microgranules, which reduced the Ag^+^ release. No effect of Ga^3+^ ions on the antibacterial activity of the samples was observed under the test conditions. This could have been caused by the lower antibacterial activity of the Ga ions or their slower release from the samples, in comparison with the Ag-enriched samples.

The agar plate test has limitations, which are related to the restricted release and migration of antibacterial ions in viscous and solid agar medium. Therefore, to gain a deeper insight into the antibacterial properties of the tested samples, an antibacterial activity assessment of porous materials and a bacterial adhesion test were performed. Due to the different form of the samples (powders and granules), the tests were performed separately for the powders pressed into the tablets and for the porous granules.

#### 2.5.2. Antibacterial Activity of Powders

The assessment of the antibacterial activity of the tested materials is based on a comparison of the number of viable bacteria eluted from the pure hydroxyapatite and Ag- and Ga-doped hydroxyapatite. The results of this assessment for pressed Ag-HA and Ga-HA powders were compared with those of the pressed HA powder and the positive controls (amount of bacteria in 50 µL of working bacterial suspensions). The tablets pressed from HA powder served as a reference, which allowed us to estimate the amount of viable bacteria eluted from pure non-doped material. As shown in Figure S2a, approximately 3% and 48% of bacteria introduced into the tablets were eluted from the HA reference samples inoculated with *S. aureus* and *S. epidermidis*, respectively. No viable Gram-positive bacteria were eluted from the Ag-HA and Ga-HA tablets (in the case of *S. epidermidis*, the results were statistically different), suggesting the antibacterial activity of both silver- and gallium-doped hydroxyapatite. However, for both Gram-negative strains, no bacteria were eluted from the HA reference tablets or from the Ag-HA and Ga-HA tablets (Figure S2a). Thus, the evaluation of antibacterial activity of the Ag and Ga ions was impossible in the case of the *E. coli* and *P. aeruginosa* strains. This observation was possibly caused by the relatively high density and low porosity of the pressed tablets, which enabled the bacteria to enter into the tablets but did not allow them to be eluted after the incubation. In turn, the bacterial adhesion test allows us to evaluate the ability of tested materials to prevent the adhesion of bacteria, which is a crucial starting point in the biofilm formation process. In this test, the tablets of pressed HA powder served as a reference for the Ag-HA tablets and the Ga-HA tablets, similar to the AATCC test method 100-2004. The results are presented in Figure S2b. For all the tested bacterial strains, Ag-HA significantly reduced the number of adhered bacteria in comparison with pure HA by 10–82%, depending on the strain (Figure S2b). A higher rate of bacterial viability reduction was observed for both Gram-negative strains (by 49–82% compared with pure HA) in comparison with both Gram-positive strains (by 10–33% compared with pure HA) (Figure S2b). The effect of gallium ions doping on the antiadhesive properties of HA was less distinct, which is probably related to the lower rate of Ga^3+^ release from the Ga-HA tablets. A statistically significant reduction in the number of adhered bacterial cells in the case of the Ga-HA tablets was found for all strains (by 12.5–64% compared with pure HA), with the exception of *S. epidermidis* (Figure S2b).

**Figure 5:**
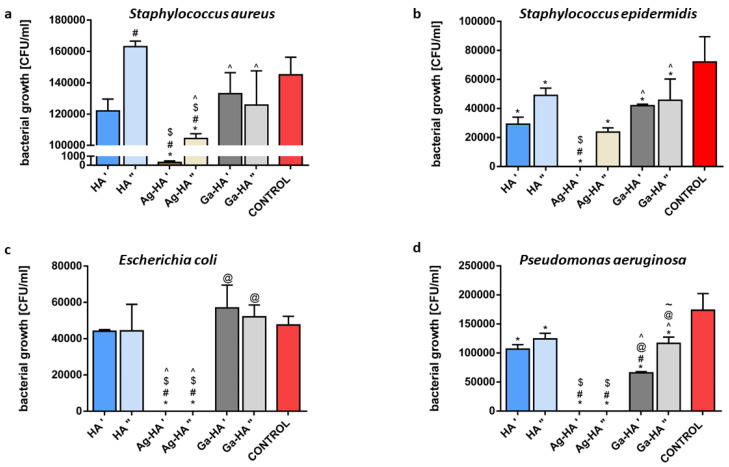
Antibacterial activity of hydroxyapatite powders against 4 bacterial strains: Staphylococcus aureus (**a**), Staphylococcus epidermidis (**b**), Escherichia coli (**c**) and Pseudomonas aeruginosa (**d**). (ʹ) and (ʺ) symbols accompanying powders designation on X axes indicate their concentration in mixtures (0.1 mg/mL and 1 mg/mL, respectively). (*) symbol indicates statistically significant differences between the samples and control, (#) symbol indicates statistically significant results between HAʹ and the samples, ($) symbol indicates statistically significant results between HAʺ and the samples, (^) symbol indicates statistically significant results between Ag-HAʹ and the samples, (@) symbol indicates statistically significant results between Ag-HAʺ and the samples, (~) symbol indicates statistically significant results between Ga-HAʹ and the samples; according to one-way ANOVA with post-hoc Dunnett’s test or post-hoc Tukey’s test (*p* < 0.05).

Difficulties in the interpretation of the results of antibacterial activity assessment for the reference and ions-doped HA powders (as shown in Figure S2a) necessitated the testing of the antibacterial activity of the powders in another mode. Thus, powdered samples were subjected to additional tests, based on the direct contact between the powders and bacteria, followed by an evaluation of their survival rate. In this test, the impact of bacterial adhesion on the tested powder was eliminated, as both free and powder-adhered cells were plated and counted. Two concentrations of powder suspensions, 0.1 mg/mL and 1 mg/mL, were tested, while the final titre of all bacterial strains was 3.0 × 10^6^ CFU/mL. A statistically different antibacterial effect was found even for pure HA powder in both tested concentrations for *S. epidermidis* and *P. aeruginosa*, causing the reduction in bacterial viability to 40–60% of the control. However, the effect of HA powder for *S. aureus* and *E. coli* was not detected (Figure 5).

Although it was recognized that hydroxyapatite was biologically inert and did not act toxically on bacteria, there were some observations indicating the antibacterial activity of nanohydroxyapatite, both artificial (obtained by the wet precipitation method or by the microwave-assisted method) [50,51] and natural source-derived [52]. Our results are, to some extent, confirmation of previous reports. The effect of the Ag-HA powder was the most striking; in the case of all strains, 1 mg/mL of Ag-HA powder reduced the bacterial viability completely or almost completely. The same was observed for the 0.1 mg/mL Ag-HA powder concentration by comparison with both Gram-negative strains, while in the case of the Gram-positive strains, the reduction in bacterial viability reached 25–35% of the control (Figure 5). The statistical analysis also confirmed that the presence of Ag ions caused a statistically different antibacterial effect in comparison with pure HA powder (with the exception of a 0.1 mg/mL concentration, tested against *S. epidermidis* strain). In turn, the effect of Ga ions in the HA powder was much less obvious. It did reduce the viability of *S. epidermidis* and *P. aeruginosa* by comparison with the control (to approx. 40% of the control) (Figure 5b,d). However, in the case of 1 mg/mL of Ga-HA and *P. aeruginosa,* the antibacterial effect was statistically different compared with the same concentration of HA powder (Figure 5d). Moreover, this effect was dose-dependent (Figure 5d). This observation is the more important in light of the fact that *P. aeruginosa* is a critically dangerous bacterial strain, causing morbidity and mortality in many patients and is remarkably resistant to antibacterial agents, thus is difficult to eradicate [53]. These observations are in accordance with the results of the bacterial adhesion test, which showed that *P. aeruginosa* is the most susceptible to Ga-HA antibacterial activity among all tested strains. To summarize, the direct contact test revealed more details than the antibacterial activity test, based on the AATCC test method 100-2004, and confirmed the strong antibacterial activity of Ag-HA and the moderate antibacterial activity of Ga-HA.

#### 2.5.3. Antibacterial Activity of Granules

**Figure 6:**
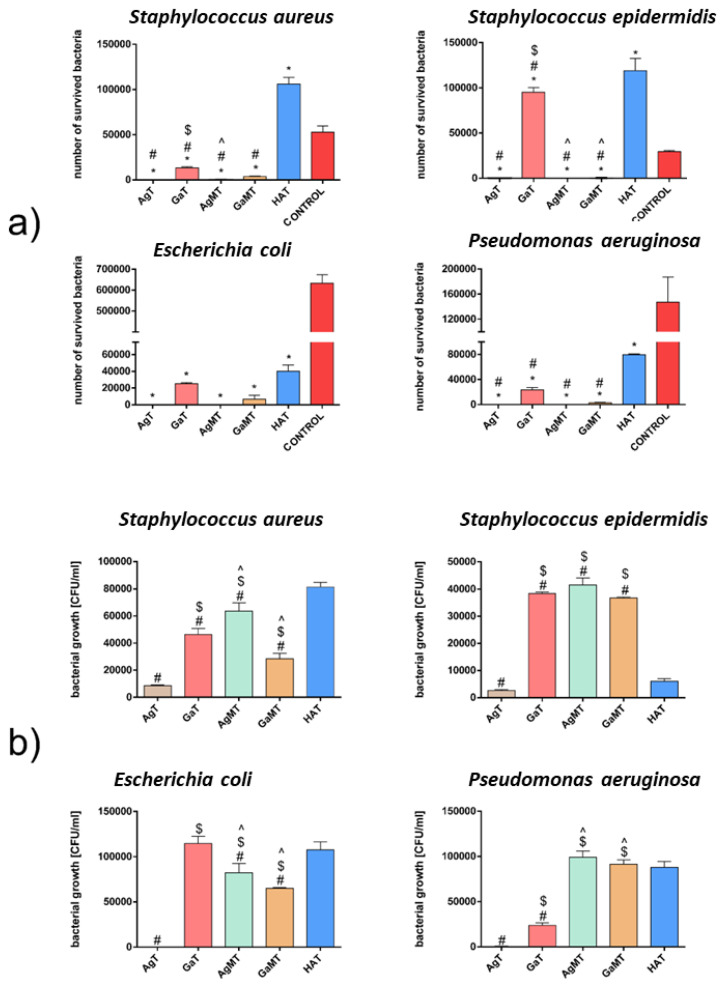
Antibacterial activity according to the AATCC 100-2004 test method (**a**) and bacterial adhesion (**b**) of hydroxyapatite-based granules against 4 bacterial strains. (*) symbol indicates statistically significant differences between the samples and control, (#) symbol indicates statistically significant results between the samples and HAT granules, ($) symbol indicates statistically significant results between AgT and all doped samples, (^) symbol indicates statistically significant results between GaT and all doped samples; according to one-way ANOVA with post-hoc Dunnett’s test or post-hoc Tukey’s test (*p* < 0.05).

For porous granules, the assessment of the antibacterial activity required a reference sample in the form of granules prepared from pure HA powder (HAT), which was prepared in an analogous way as AgT, GaT, AgMT and GaMT. The physicochemical properties of the HAT granules (size, morphology, porosity) were similar to the AgT and GaT granules (data not shown). The results of the antibacterial activity for these granules, based on the AATCC test method 100-2004, showed that amount of both Gram-positive bacteria significantly increased after incubation with the reference HAT granules, in comparison with the control (Figure 6a).

This phenomenon might have been caused by the presence of organic polymers (sodium alginate and chondroitin sulphate), which served as a nutrient for bacterial cell propagation. Alginate oligosaccharides are known for their antibacterial activity exhibited in relation to *S. aureus* and other staphylococci [54]. However, hydrogel wound dressings, based on alginate polymers, require additional antibacterial agents to reveal antibacterial activity [55]. In turn, the number of *E. coli* and *P. aeruginosa* was reduced after incubation with HAT, which can especially be explained in the case of the latter strain, as *P. aeruginosa* cannot use alginate as a carbon source [56]. Silver-containing AgT and AgMT granules caused the most significant mortality of all the tested strains (Figure 6a), confirming their strong antibacterial activity. Gallium-doped materials (GaT and GaMT) also caused a decrease in bacterial viability by 70–100% by comparison with pure HAT (Figure 6a); only in the case of *S. epidermidis* and GaT granules was this effect less notable (only a 20% decrease). The bacterial adhesion test related to the second aspect of the bacteria-biomaterial interactions showed the strong impact of AgT on bacterial adhesion (although for *S. epidermidis*, it was less and was not significant) (Figure 6b). AgMT granules exhibited a much weaker effect on bacterial adhesion than AgT, which can be explained by the lower content of silver in the granules. The effect of gallium presence in GaT and GaMT on bacterial adhesion limitation was much less distinct than that observed for the presence of silver in the tested granules (Figure 6b). Surprisingly *E. coli* showed a stronger reaction to GaMT than to GaT, although the latter contained a higher Ga concentration (Figure 6b).

An unexpected observation was made in the case of *S. epidermidis*, namely, the bacterial adhesion to AgMT, GaT and GaMT was significantly higher than that of the reference HAT granules (Figure 6b). This phenomenon is difficult to explain; however, this may be on account of the different surface topography of these granules.

AgM and GaM microgranules, due to the low porosity resulting from the high sintering temperature (15–25 µm and 5 µm; as mentioned above) and micro-dimensional form, could not be evaluated for antibacterial activity, using the same tests as other materials. Therefore, extracts obtained after microgranule incubation (24 h) with bacterial culture broth were inoculated with four reference bacterial strains. Then, the bacterial growth in the extracts was monitored. However, no antibacterial effect was observed. The rate of bacterial growth in both collected extracts was on a comparable level with the bacterial growth of the control (data not shown). The above results may be explained by the low porosity of microgranules, which negatively affects the release of Ag^+^ and Ga^+^ ions from the samples in this particular test. However, we have indirect proof that AgM and GaM microgranules reveal antibacterial activity, namely, that AgMT and GaMT granules (composed of 50% of AgM and GaM microgranules, respectively) exhibit notable antibacterial activity, according to the AATCC 100-2004 test method (Figure 6a). Therefore, their antibacterial activity must have been related to the presence of AgM and GaM microgranules in the granules’ structure.

## 3. Materials and Methods

### 3.1. Synthesis of Silver- or Gallium-Containing Hydroxyapatite Powders

Hydroxyapatite powders enriched with gallium (Ga-HA) or silver ions (Ag-HA) with 0.45 wt% and 0.54 wt% nominal value of Ga or Ag, respectively, were synthesized using the conventional wet method (coprecipitation in an aqueous solution), which was described in detail in our previous work [57]. The following reagents were used in the aforementioned synthesis: calcium nitrate tetrahydrate Ca(NO_3_)_2_·4H_2_O (Sigma-Aldrich, Bangalore, India), ammonium dibasic phosphate (NH_4_)_2_HPO_4_ (Chempur, Piekary Śląskie, Poland), silver nitrate AgNO_3_ (Avantor Performance Materials, Gliwice, Poland) and gallium nitrate trihydrate Ga(NO_3_)_3_·3H_2_O (Sigma-Aldrich, Burlington, MA, USA) as sources of calcium, phosphorus, silver and gallium, respectively. Briefly, an aqueous solution of (NH_4_)_2_HPO_4_ was added dropwise into an aqueous solution of Ca(NO_3_)_2_·4H_2_O and one of the aforementioned reagents (AgNO_3_ or Ga(NO_3_)_3_ 3H_2_O) and stirred gently at pH 10 and at a temperature of 60 °C for 2 h. Then, the obtained precipitate was left for 24 h for ageing. Next, the precipitates were filtered, soaked several times in distilled water and dried at 120 °C in air. For comparison, pure, unsubstituted hydroxyapatite (HA) was synthesized by the same method.

### 3.2. Preparation of Microgranules

Microgranules were obtained using Ag-HA and Ga-HA powders according to the method adapted from [58]. Additional reagents, used in the preparation of microgranules by using camphene emulsion, were as follows: gelatine, 20 mesh pure p.a. (Chempur, Piekary Śląskie Poland); poly(vinyl alcohol) PVA, average molecular weight 130,000 (Sigma-Aldrich, USA); poly(acrylic acid sodium salt) (Sigma-Aldrich, St. Louis, MO, USA); Triton™ X-100 (Sigma-Aldrich, St. Louis, MO, USA); camphene (Sigma-Aldrich, Madrid, Spain).

Firstly, a 10% aqueous gelatine solution was prepared, with the addition of 2% PVA. Afterwards, 0.2% Triton X-100 and 0.3% poly(acrylic acid sodium salt) were added to the solution as dispersants. Meanwhile, camphene and Ag-HA or Ga-HA powder were mixed together at 60 °C in a 0.5:1 ratio. A 10% gelatine solution was added to the camphene/Ag-HA (Ga-HA) mixture (2 mL of solution per 1 g of hydroxyapatite powder) and the obtained slurry was dispersed in oil in a beaker using a magnetic stirrer (150–250 rpm). Subsequently, the beaker was kept in an ice-cooled bath for 5 min, then the obtained microgranules were separated from the oil, rinsed with ethanol and finally dried at room temperature.

At the next stage, microgranules were sintered at a high temperature (initially 500 °C for 1 h at a heating rate of 3 °C/min, then at 1250 °C for 3 h at a heating rate of 5 °C/min). The granules were then sieved using test sieves (Ø 0.2 mm and Ø 1 mm), in order to separate those with a 0.2–1 mm diameter.

The obtained granules were named AgM and GaM for silver- and gallium-containing samples, respectively. For comparison, pure HA was synthesized according to the aforementioned procedure.

### 3.3. Preparation of Composite Granules

In order to fabricate composite granules, the following reagents were used: sodium alginate (Sigma Aldrich, US), chondroitin sulphate sodium salt (TCI, Belgium), calcium chloride anhydrous CaCl_2_ (Sigma-Aldrich, China) and the following previously synthesized powders and microgranules: HA, Ag-HA, Ga-HA, AgM and GaM.

At first, a 4% aqueous sodium alginate solution was prepared at 40 °C and chondroitin sulphate sodium salt was added to obtain a 0.5% suspension. Then, two types of composite granules were prepared.

In the first type of granules, 1 g of Ag-HA or Ga-HA (or HA) powder was added to the suspension (10 mL) and mixed vigorously, resulting in a milky, dense slurry. Meanwhile, the cross-linking solution (1.5% CaCl_2_) was prepared. Finally, the slurry was added dropwise to a CaCl_2_ solution, stirred using a magnetic stirrer and granules were formed. The granules obtained were left in the cross-linking agent for 10 min, rinsed with distilled water, dried in air and then lyophilized.

During the preparation of the second type of granules, pure, unsubstituted HA and AgM or GaM microgranules (ratio 1:1) were used instead of Ag-HA or Ga-HA powders. The other stages of production remained unchanged. All the obtained granules are listed in Table 6.

### 3.4. Physicochemical Analysis of Ag-HA and Ga-HA

The phase composition of the powder samples was determined by powder X-ray diffractometry (PXRD, Bruker D8 Advance, Billerica, MA, USA). The diffractometer was equipped with a LYNEXEYE position sensitive detector and with Cu-Kα radiation (λ = 0.15418 nm). The measurements were carried out in the Bragg–Brentano (θ/θ) horizontal geometry (flat reflection mode) between 15° and 60° (2θ) in a continuous scan, using 0.03° steps and 2 s/step (total time 384 s/step). Phase identification was achieved by comparing the obtained diffractograms of HA, Ag-HA and Ga-HA samples with the JCPDS 09-0432 standard pattern.

Fourier-transform infrared studies (FT-IR) were conducted using the Spectrum 1000 spectrometer (Perkin Elmer, Llantrisant, UK). The data were collected with a 2 cm^−1^ resolution over a range of 4000–400 cm^−1^ at 30 scans, using the standard KBr pellet technique.

PXRD patterns and FT-IR spectra were processed using GRAM/AI 8.0 software (Thermo Scientific, Burlington, ON, USA) and subsequently, graphs were prepared with KaleidaGraph 3.5 software (Synergy Software, Reading, PA, USA).

The gallium and silver content in the Ga-HA and Ag-HA samples, respectively, was measured by atomic absorption spectrometry (AAS). Briefly, AgNO_3_ (Avantor Performance Materials, Poland) and Ga(NO_3_)_3_·3H_2_O (Sigma-Aldrich, USA) were weighed out, dissolved in distilled water (separately) and then diluted several times, in order to prepare the solutions necessary for the calibration curves. Then, the known quantities of synthesized Ag-HA and Ga-HA powders were weighted out, dissolved in suprapure 63% HNO_3_ and adequately diluted with distilled water. Finally, the obtained solutions were measured by AAS spectrometry (ANALYST 400, Perkin Elmer, Llantrisant, UK), with detection at a wavelength λ = 328.07 nm for silver and λ = 287.42 nm for gallium.

### 3.5. Physicochemical Analysis of the Granules

In order to determine the morphology of the prepared granules, a microscopical study was performed using scanning electron microscopy (SEM) JSM 6390 LV (JEOL, Tokyo, Japan) at 20 or 30 kV accelerating voltage. The survey was based on taking images of granules (previously covered with a gold layer in a vacuum chamber) from both the outer and the inner surface (after the cross-section). The cross-sections were prepared by carefully cutting the granules with a surgical lancet.

The porosity and specific surface area of the granules were evaluated using the mercury intrusion porosimetry method with the Autopore IV 9510 instrument (Micromeritics, Norcross, GA, USA). The measurements were conducted with Hg intrusion pressure in the range of 0.0035–400 MPa. The dried fragments of the tested sample were degassed in a penetrometer to a pressure of 50 mmHg. Finally, the volumes and size distributions of the pores were calculated using the Washburn equation [59], which is as follows:

(3) $$P_{C}=\frac{2\sigma \cos\theta }{r}$$

where

*P_C_*—capillary pressure
*σ*—mercury interfacial tension
*θ*—contact angle
*r*—pore radius.

The samples were also tested in order to evaluate the mechanical compressive strength, by measuring the strength needed for destruction of the granules. The study was performed using the Tinius Olsen H 10K-S instrument (Tinius Olsen, Horsham, PA, USA). Briefly, the granules were placed between the stationary plate and the measuring head, then were put under a pressure test, while moving the head at a speed of 5 mm/s. The mechanical compressive strength is a ratio of pressure used for the destruction of the granule (N) and the diameter of the granule (mm).

In order to evaluate in vitro Ga^3+^ and Ag^+^ release from the granules, 0.5 g of each sample was placed in a conical tube with a volume of 50 mL, then 50 mL of phosphate buffered saline (PBS) of pH = 7.4 was added. Afterwards, the tubes were placed in a water bath at 37 °C and stirred gently. The release study of silver and gallium ions was carried out for three weeks. Sample aliquots of 10 mL were taken at specific time intervals, namely 1 h, 2 h, 3 h, 6 h, 12 h, 24 h, 2 days, 5 days, 1 week, 2 weeks and 3 weeks, and replaced with the same amount of fresh PBS.

The amount of released ions was determined by inductively coupled plasma mass spectrometry (ICP-MS), using an ICP mass spectrometer, Thermo Electron X Series II (Thermo Electron Corporation, Allentown, PA, USA).

### 3.6. In Vitro Cytotoxicity Studies

In order to evaluate the cytotoxicity to mammalian cells, the materials (Ag-HA, Ga-HA powders and six types of granules), were tested with the following two assays: the neutral red uptake (NRU) test performed on the basis of the ISO 10993 guideline Annex A [60] and the MTT assay, based on the reduction in 3-(4,5-dimethylthiazol-2-yl)-2,5-diphenyltetrazolium bromide by the mitochondrial succinate dehydrogenase. In the NRU assay, the quantitative estimation of viable cells in the tested cultures was based on their neutral red uptake in comparison to the results obtained for untreated cells. Dead cells have no ability to accumulate the dye in their lysosomes. The MTT assay allowed us to evaluate the mitochondrial metabolic activity of the tested cultures. Only cellular oxidoreductase enzymes in living cells have the ability to reduce the tetrazolium MTT dye into an insoluble, purple formazan. Both tests were performed with the BALB/c 3T3 clone A31 mammalian cell line (mouse embryonic fibroblasts from American Type Culture Collection).

For the NRU and MTT assays, the BALB/c 3T3 cells were seeded in 96-well microplates (15,000 cells/100 µL) in DMEM (Lonza) culture medium (supplemented with 10% of calf bovine serum, 100 IU/mL penicillin and 0.1 mg/mL streptomycin) and incubated for 24 h (5% CO_2_, 37 °C, >90% humidity). At the end of the incubation, each well was examined under a microscope to ensure that the cells formed a confluent monolayer. Subsequently, the culture medium was replaced by the tested extracts of materials. The extracts were prepared by incubation of the tested materials in the cell culture medium (100 mg/mL) with reduced serum concentration (5%) at 37 °C for 24 h, then shaken and sterilized by filtration. The cells were treated with four dilutions of each extract in a twofold dilution series for 24 h (three data points for each). Subsequently, the treatment medium was removed. The cells were washed with PBS and treated with the neutral red medium or MTT medium for 2 h. Then, the medium was discarded, the cells were washed with PBS and treated with desorbing fixative (ethanol and acetic acid water solution or isopropanol). The amount of neutral red medium accumulated by the cells was evaluated colorimetrically at 540 nm. The amount of insoluble purple formazan was evaluated colorimetrically at 570 nm. Polyethylene film and latex were used as the reference materials (with no cytotoxicity and high cytotoxicity, respectively). The percentage of viable cells in each well was calculated by comparing its OD_540_ or OD_570_ result with the mean result obtained for untreated cells (incubated in the same conditions with fresh culture medium). Samples were considered cytotoxic if they reduced cell survival or mitochondrial metabolic activity below 70%, compared to the untreated cells (a baseline cell viability and enzymatic activity). When the BALB/c 3T3 cell viability was not decreased below 70% across the whole range of tested dilutions of the samples, it was considered non-cytotoxic in this range of concentrations.

### 3.7. Antibacterial Activity Studies

#### 3.7.1. Strains and Maintenance

The following bacterial strains from ATCC were used in this study: *Staphylococcus aureus* ATCC 25923, *Staphylococcus epidermidis* ATCC 12228, *Pseudomonas aeruginosa* ATCC 27853 and *Escherichia coli* ATCC 25922. Bacteria, maintained in microbanks at −80 °C, were cultured in Mueller–Hinton (M–H) agar medium (Biomaxima, Lublin, Poland), at 37 °C for 20–24 h and then transferred into a Mueller–Hinton (M–H) broth (Biomaxima, Lublin, Poland) for a further 24 h incubation at 37 °C. Depending on the particular experiment, bacterial inoculates were obtained either by bacterial cell scrapping from agar medium to sterile saline or by the dilution of the suspension in the culture broth prior to the experiments. Bacterial titres were selected individually for each test. The tested biomaterials were sterilized by EthO (ethylene oxide) before the tests. All tests of antibacterial activity were performed in triplicate.

#### 3.7.2. Agar Plate Test

Wells (Ø = 7.5 mm) were drilled in Mueller–Hinton agar (Biomaxima, Lublin, Poland) in 90 mm Petri dishes (thickness: approx. 5 mm). A number of 25 ± 1 mg samples, sterilized in EtOh, were placed inside the wells to evenly cover the surface of the wells. Then, liquid Mueller–Hinton agar medium (approx. 40 °C) was poured into the wells and allowed to set. Next, 100 µL of bacterial inoculate in sterile 0.9% NaCl (0.1 McFarland standard, an equivalent of approx. 3.0 × 10^7^ CFU (colony forming units)/mL) was placed onto the agar and evenly spread. The test was performed individually for each bacterial strain. Subsequently, plates were incubated at 37 °C for 20–24 h. Afterwards, bacterial growth inhibition zones (in mm) were measured.

#### 3.7.3. Antibacterial Activity Test (AATCC Test Method 100-2004 “Antibacterial Finishes on Textile Materials: Assessment of Developed from American Association of Textile Chemists and Colorists”)

Pellets for antibacterial activity studies were obtained by weighting 0.2 g of powder (HA, Ag-HA or Ga-HA) and forming it into a tablet, using a manual hydraulic pellet press. An evaluation of the antibacterial activity of the tested samples was performed on the basis of the AATCC test method 100-2004 for textile materials and adapted for porous ceramic materials. Briefly, samples (200 ± 15 mg of pellets formed from the obtained powders and 50 ± 2 mg of each synthesized granule) were sterilized by ethylene oxide. AgM and GaM microgranules were not subjected to this test, due to their small size and low porosity caused by high sintering temperature, which significantly reduced the accuracy of this particular measurement. A working bacterial suspension (3.0 × 10^7^ CFU/mL) of each strain was prepared in M–H broth and diluted 250-fold in sterile 0.9% NaCl. Samples were placed on sterile microscope slides (Chemland, Stargard Szczeciński, Poland) and inoculated with a working bacterial suspension, which was completely absorbed by the samples, leaving no remaining liquid (50 µL both for 200 mg tablets and 50 mg granule portions). Then, all samples were transferred to sterile 50 mL Falcon tubes (Corning, Union City, CA, USA), which were screwed to prevent evaporation and then incubated at 37 °C for 24 h. An amount of 50 µL of working bacterial suspension of each strain was placed in another sterile 50 mL Falcon tube and treated as above, to control the viability of bacteria, without contact with the tested materials (control+). Afterwards, 5 mL of sterile 0.9% NaCl was added to all samples and vigorously shaken (1 min) to elute the bacterial cells. Samples of the collected eluate were plated onto M–H agar Petri dishes using an EasySpiral Dilute (Interscience, Saint Nom La Bretèche, France) automatic plater (each sample in triplicate). M–H agar plates with plated bacteria eluted from the samples were incubated at 37 °C for 20–28 h. CFUs were then counted for each plate using a Scan 300 colony counter (Interscience, Saint Nom La Bretèche, France). The reduction in the number of bacteria was calculated as a percentage of CFU in control+ for each bacterial strain individually.

#### 3.7.4. Bacterial Adhesion Test

The samples (200 ± 15 mg of pellets formed from the obtained powders and 50 ± 2 mg of each synthesized granule) were sterilized by ethylene oxide in the wells of a 12-well plate (Corning, Union City, CA, USA). AgM and GaM microgranules were also not subjected to this test for the same reason as mentioned in Section 3.7.3, namely due to the difficulty in the test performance. Then, 2 mL of bacterial suspensions (3.0 × 10^8^ CFU/mL) in M–H broth were added to each well individually for each bacterial strain. Next, the plates were protected with stripes of parafilm (Bemis, Neenah, WI, USA) to prevent evaporation at the liquid phase, then incubated in an Innova 42 incubator (New Brunswick Scientific, Enfield, CT, USA) at 37 °C, 2 h, 100 rpm. Afterwards, tablets and granules were aseptically transferred onto a sterilized Whatman filter membrane to remove the excess of the bacterial suspension, placed in sterile 50 mL Falcon tubes (Corning, Union City, CA, USA) and washed carefully 4 times with 20 mL of 0.9% NaCl, to remove all unbound bacterial cells. Then, the pieces were transferred again to the sterilized Whatman filter membrane to remove excess saline, moved to another set of 50 mL Falcon tubes and treated with 1 mL of 0.25% trypsin-EDTA solution (Sigma-Aldrich, St. Louis, MO, USA) to digest the proteins, which enable the cells to adhere to the prostheses (15 min at 37 °C). After intense vortexing (1 min), trypsin was inactivated with 4 mL of M–H broth and diluted with 5 mL of 0.9% NaCl. Finally, 50 µL of each resulting liquid was plated in triplicate on M–H agar plates using the EasySpiral Dilute plater (Interscience, Saint Nom La Bretèche, France), then the plates were incubated at 37 °C for 20–24 h and the CFU were counted on each plate using a Scan 300 counter (Interscience, Saint Nom La Bretèche, France).

#### 3.7.5. Direct Contact with Powders Test

Sterilized HA, Ag-HA and Ga-HA powders were suspended in sterile PBS pH 7.4 to obtain the suspensions 2 mg/mL and 0.2 mg/mL, which were sonicated (Sonic-6, Polsonic, Warsaw, Poland) for 15 min for better dispersion. Bacterial working solutions were prepared for each bacterial strain, including 6.0 × 10^6^ CFU/mL of M–H broth diluted 125-fold in sterile 0.9% NaCl. Then, the powder suspensions and bacterial working solutions were mixed in sterile 5 mL screwed tubes in the proportion 1:1 (resulting in a mixture of 1 mg/mL or 0.1 mg/mL of powder with 3.0 × 10^6^ CFU/mL in M–H broth, diluted 250-fold in sterile 0.9% NaCl). Positive controls (control+) for each bacterial strain were prepared by mixing PBS pH 7.4 and bacterial working solutions under the same conditions as the powder suspensions. The tubes were then incubated at 37 °C, for 24 h at 5 rpm using a RM 5–30 V CAT roller mixer (Ingenieurbüro CAT M.Zipperer, Ballrechten-Dottingen, Germany). Finally, samples of the mixtures (50 µL) were plated onto M–H agar Petri dishes using an EasySpiral Dilute (Interscience, Saint Nom La Bretèche, France) automatic plater (each sample in triplicate). M–H agar plates with plated bacteria eluted from the samples were incubated at 37 °C for 20–28 h. CFUs were then counted for each plate, using a Scan 300 colony counter (Interscience, Saint Nom La Bretèche, France).

#### 3.7.6. Antibacterial Activity in Sample Extracts

Samples of AgM and GaM microgranules were sterilized by ethylene oxide in 50 mL Falcon tubes and were then immersed in sterile PBS pH 7.4 (proportion: 0.1 g of granules/1 mL PBS) at 37 °C for 24 h in an Innova 42 incubator (New Brunswick Scientific, Enfield, CT, USA) at 100 rpm. An amount of 100 µL of the extracts was collected under sterile conditions and transferred into the wells of a 96-well plate, mixed with 100 µL of sterile M–H broth and inoculated with 10 µL of bacterial working suspension to produce a final titre of 0.75 × 10^5^ CFU/mL. An amount of 100 µL PBS with pH 7.4, mixed with 100 µL of sterile M–H broth and inoculated with bacteria as above, served as a positive control, while the non-inoculated variant served as an assay control. Then, the plates were incubated at 37 °C and 200 rpm for 24 h in an Innova 42 incubator (New Brunswick Scientific, Enfield, CT, USA) and absorbance of the extracts was measured at 660 nm using a Synergy H4 Hybrid Microplate Reader (Thermo Electron Corporation, Allentown, PA, USA). The absorbance of the assay control was subtracted from the absorbance of the samples.

#### 3.7.7. Statistical Analysis

Statistically significant differences between the various samples were calculated according to a one-way ANOVA with post-hoc Dunnett’s test or post-hoc Tukey’s test, or according to a Student’s *t*-test, using GraphPad Prism 8.0.0 Software (San Diego, CA, USA). Samples were used in different numbers for various tests but at least in triplicate (details in appropriate sections).

## 4. Conclusions

In our work, we investigated the antibacterial activity of various materials with potential application as bone substitutes. We focused on two ionic dopants with antibacterial properties, Ag^+^ and Ga^3+^, due to their different mechanisms of action and efficiency.

Three types of granules containing silver or gallium ions (ceramic microgranules, hydroxyapatite/alginate composite granules, and granules made of ceramic microgranules and alginate) were examined. The studied samples differed in morphology, porosity and mechanical properties. The granules were tested for the release of silver and gallium ions and for their antibacterial properties. It should be noted that the granules exhibited different silver and gallium release. Two cytotoxicity tests showed that the majority of the materials are not toxic, except the Ag-HA powder and AgT granules that were found toxic in both assays. However, in the first of the dilutions in the twofold dilution series, none of these samples negatively affected the cell culture condition. The results of the antibacterial tests turned out to be promising, as all the silver-containing materials caused significant mortality of the tested bacterial strains. The antibacterial efficacy of gallium-containing materials were significantly lower.

The aim of our future work is to investigate the porous granules doped with silver or gallium as antibiotic delivery systems targeting bone tissue.

We assume that the presence of silver or gallium ions, together with an antibiotic, may improve the effectiveness of the prevention and treatment of intraoperative infections of osseous tissue.

## Figures and Tables

**Table 1:** Parameters of the obtained HA powders.

	Ag-HA	Ga-HA
**Crystallinity index (CI)**	0.45	0.39
**Crystallite size—*c*-axis (nm)**	27 ± 2	26 ± 3
**Crystallite size—*a*-axis (nm)**	6 ± 2	7 ± 2
**Ionic dopant content (wt%)**	0.46	0.41

**Table 2:** Results of porosity and mechanical strength studies.

Sample	Total Volume of Pores (cm^3^/g)	Porosity of Granules (%)	SSA of Pores (m^2^/g)	Volume of Mesopores (cm^3^/g)	Percentage of Mesopores (%)	Average Diameter of Pores (nm)	Apparent Density of Granules (g/cm^3^)	Average Mechanical Strength (N/granule)
**GaT**	0.87	65	79	0.32	37	40	0.8	34
**GaMT**	0.28	36	21	0.06	22	35	1.3	110
**AgT**	0.94	65	55	0.23	25	65	0.7	37
**AgMT**	0.56	55	42	0.12	22	45	1.0	15

**Table 3:** Results of the neutral red uptake test for the highest concentrations of tested extracts (100 mg/mL) in comparison to the untreated control.

Sample	Cells Viability ± SD (%)	IC50 (mg/mL)	Classification
**HA powder**	101 ± 2	N	Non-cytotoxic
**Ag-HA powder**	**0 ± 0**	75	Cytotoxic
**Ga-HA powder**	80 ± 10	N	Non-cytotoxic
**AgM granules**	100 ± 3	N	Non-cytotoxic
**GaM granules**	98 ± 4	N	Non-cytotoxic
**AgT granules**	**0 ± 0**	61	Cytotoxic
**GaT granules**	80 ± 4	N	Non-cytotoxic
**AgMT granules**	93 ± 7	N	Non-cytotoxic
**GaMT granules**	96 ± 5	N	Non-cytotoxic
**LT**	0 ± 0	<10	Cytotoxic
**PE**	102 ± 7	N	Non-cytotoxic

LT—latex, reference cytotoxic material. PE—polyethylene foil, reference non-cytotoxic material. N—calculation was not possible due to the lack of cytotoxicity in the whole range of tested concentrations. SD—standard deviation. Results with cells viability decreased under 70% are bolded.

**Table 4:** Results of the MTT assay for the highest concentrations of tested extracts (100 mg/mL) in comparison to the untreated control.

Sample	Cells Viability ± SD (%)	IC50 (mg/mL)	Classification
**Ag-HA powder**	0 ± 0	59	Cytotoxic
**Ga-HA powder**	37 ± 9	86	Cytotoxic
**AgM granules**	107 ± 2	N	Non-cytotoxic
**GaM granules**	108 ± 5	N	Non-cytotoxic
**AgT granules**	17 ± 7	74	Cytotoxic
**GaT granules**	70 ± 9	N	Non-cytotoxic
**AgMT granules**	101 ± 0	N	Non-cytotoxic
**GaMT granules**	107 ± 15	N	Non-cytotoxic
**LT**	0 ± 0	<10	Cytotoxic
**PE**	102 ± 7	N	Non-cytotoxic

**Table 5:** Results of agar plate test. Diameter of the well drilled in agar medium: 7.5 mm.

Sample	Zones of Bacterial Growth Inhibition (mm)			
	*Staphylococcus* *aureus*	*Staphylococcus* *epidermidis*	*Escherichia coli*	*Pseudomonas* *aeruginosa*
**HA**	0	0	0	0
**Ag-HA**	12	13	11	12
**Ga-HA**	0	0	0	0
**AgM**	0	0	0	0
**GaM**	0	0	0	0
**AgT**	10	10	8	10
**GaT**	0	0	0	0
**AgMT**	0	0	0	0
**GaMT**	0	0	0	0

**Table 6:** List of all the obtained granules from Ag-HA or Ga-HA materials.

Granules	Fabrication method	Comment
**AgM**	Camphene emulsion	Microgranules containing Ag-HA
**GaM**	Camphene emulsion	Microgranules containing Ga-HA
**HAT**	Alginate cross-linking	Composite granules containing HA
**AgT**	Alginate cross-linking	Composite granules containing Ag-HA
**GaT**	Alginate cross-linking	Composite granules containing Ga-HA
**AgMT**	Alginate cross-linking	Composite granules containing AgM and HA
**GaMT**	Alginate cross-linking	Composite granules containing GaM and HA

## Data Availability

The data that support the findings of this study are available from the corresponding author upon reasonable request.

## Supplementary Materials

The following supporting information can be downloaded at: https://www.mdpi.com/article/10.3390/ijms23137102/s1.
