# Supplementary material for: Antibacterial and Cytotoxicity Evaluation of New Hydroxyapatite-Based Granules Containing Silver or Gallium Ions with Potential Use as Bone Substitutes
Source: Int J Mol Sci. 2022 Jun 26;23(13):7102. doi: 10.3390/ijms23137102 (PMC9266790; doi:10.3390/ijms23137102)
Supplement: Supplementary file 1 [file ijms-23-07102-s001.zip › ijms-1736351-SI.pdf]

Article: SUPPLEMENTARY MATERIALS

Antibacterial and Cytotoxicity Evaluation of New Hydroxyapatite-Based Granules Containing Silver or Gallium Ions with Potential Use as Bone Substitutes

Kamil Pajor [1], Anna Michalicha [2], Anna Belcarz [2], Łukasz Pajchel [1], Anna Zgadzaj [3], Filip Wojas [3] and Joanna Kolmas [1,*]

[1] Department of Analytical Chemistry, Chair of Analytical Chemistry and Biomaterials, Medical University of Warsaw, Faculty of Pharmacy, 02-097 Warsaw, Poland; kamil.pajor@wum.edu.pl (K.P.); lukasz.pajchel@wum.edu.pl (Ł.P.)

[2] Chair and Department of Biochemistry and Biotechnology, Medical University of Lublin, 20-093 Lublin, Poland; anna.michalicha@gmail.com (A.M.); anna.belcarz@umlub.pl (A.B.)

[3] Department of Environmental Health Sciences, Medical University of Warsaw, Faculty of Pharmacy,02-097, Warsaw, Poland; anna.zgadzaj@wum.edu.pl

* Correspondence: joanna.kolmas@wum.edu.pl

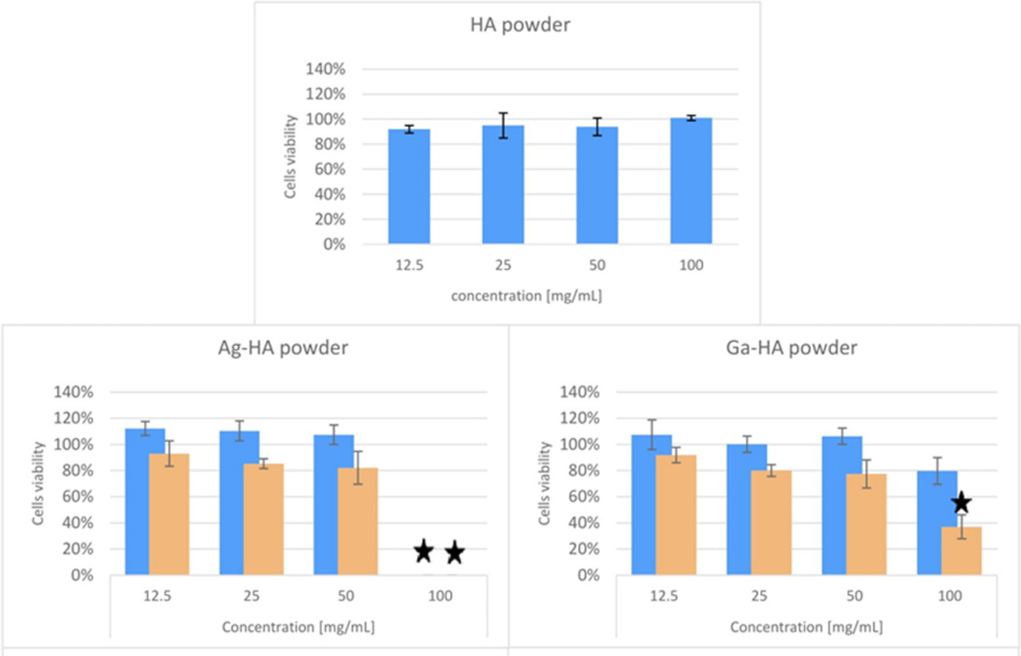

Figure S1. The NRU (blue bars) and MTT (orange bars) tests results obtained for powders in the whole range of tested extracts concentrations. Black stars indicate the decrease in the cells viability under 70%, which classifies the sample as cytotoxic.

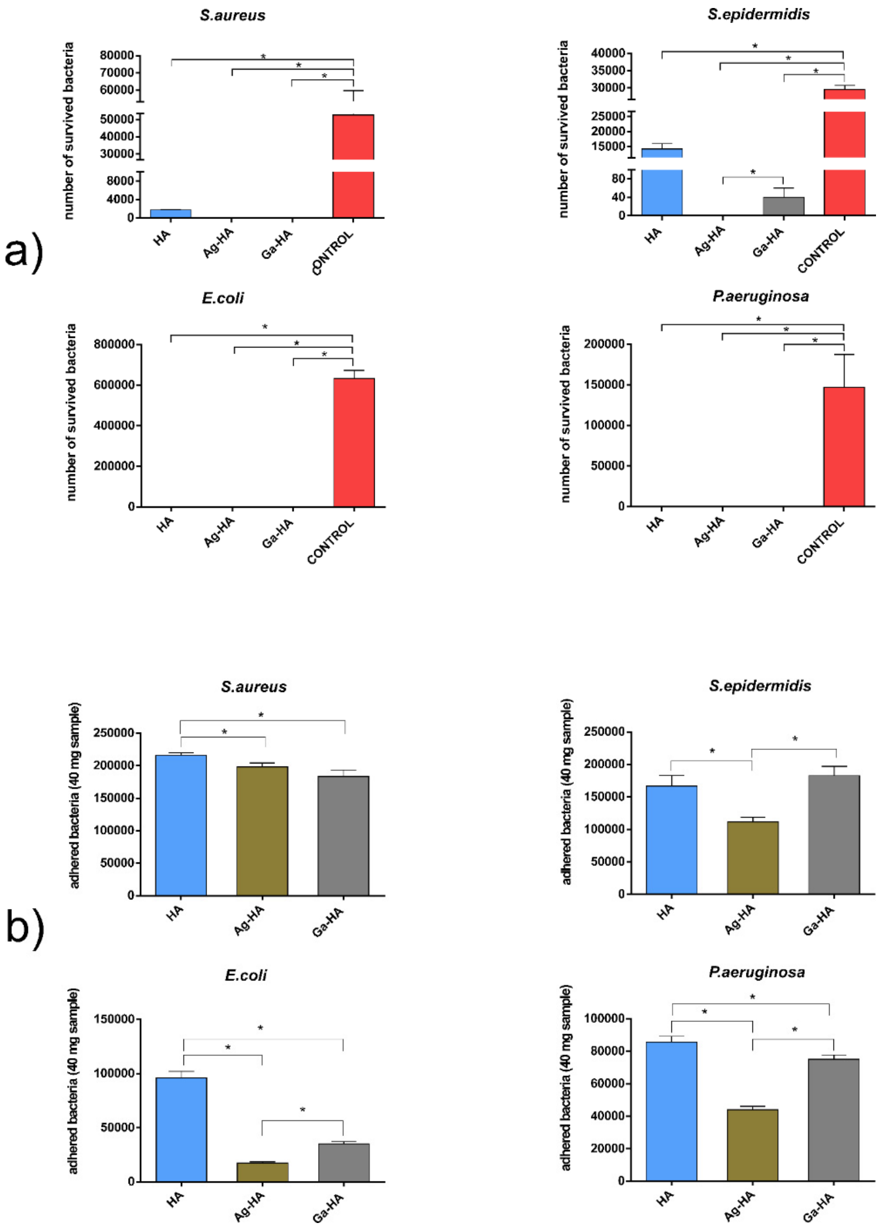

Figure S2. Antibacterial activity according to AATCC 100-2004 test method (**a**) and bacterial adhesion (**b**) of hydroxyapatite powders (pressed in tablets) against 4 bacterial strains. (*) symbol indicates statistically significant differences between samples (or controls), according to one-way ANOVA with post-hoc Dunnett's test or post-hoc Tukey's test ($p < 0.05$).
